# *MAPT*-isoform 0N3R is essential for human brain development: Loss-of-function for novel TAU-associated disease paradigms

**DOI:** 10.4103/NRR.NRR-D-25-00298

**Published:** 2025-09-29

**Authors:** Hans Zempel

**Affiliations:** 1 Institute of Human Genetics, Faculty of Medicine and University Hospital Cologne, Cologne, Germany; 2 Center for Molecular Medicine Cologne (CMMC), University of Cologne, Cologne, Germany

**Keywords:** 0N3R isoform, alternative splicing, Alzheimer’s disease, intellectual disability, neurodevelopmental disorders, TAU protein, tauopathy

## Abstract

TAU, a microtubule-associated protein, encoded by the microtubule-associated protein tau (*MAPT*) gene, is a central regulator of microtubule stability and axonal function in the human brain, with its pathological aggregation representing a hallmark of Alzheimer’s disease and related tauopathies. Despite extensive research into the role of TAU in neurodegeneration, its essentiality for human brain development has remained unclear. This perspective synthesizes recent genetic, molecular, and cellular evidence to demonstrate that the human brain-specific TAU isoform 0N3R is indispensable for proper neurodevelopment, pointing to loss-of-function of this isoform as a novel paradigm for TAU-associated disease. Alternative splicing of *MAPT* generates six brain-specific TAU isoforms, with 0N3R being exclusively expressed during fetal brain development. Analysis of large-scale human genetic datasets (gnomAD v4.0.0) reveals a high probability of loss-of-function intolerance (pLI = 0.96) for the 0N3R isoform. This is in stark contrast to the canonical Matched Annotation from the NCBI and EMBL-EBI (MANE) transcript and peripheral “Big TAU,” both of which are tolerant to loss-of-function mutations. This intolerance is further supported by the scarcity of loss-of-function mutations in 0N3R-encoding exons and high missense constraint scores, suggesting strong evolutionary selection against disruption of this isoform. Functional studies using human induced pluripotent stem cell-derived cortical neurons with CRISPR-Cas9-mediated *MAPT* knockout reveal that, unlike in murine models where compensation by other microtubule-associated proteins occurs, loss of TAU in human neurons leads to deficits in neurite outgrowth, axon initial segment shortening, and a trend toward hyperexcitability, accompanied by broad transcriptomic changes affecting genes involved in microtubule organization and synaptic structure. Remarkably, re-expression of any of the six human brain-specific TAU isoforms rescues these phenotypes, underscoring their functional redundancy during development. These findings position the 0N3R isoform as essential for human brain development and suggest that loss-of-function mutations affecting this isoform likely result in neurodevelopmental impairment, potentially manifesting as intellectual disability without overt dysmorphic features. This contrasts with the apparent tolerance to *MAPT* loss-of-function in mice and peripheral tissues, highlighting a critical species- and isoform-specific requirement for TAU in human neurodevelopment. The hypothesis of 0N3R-TAU loss-of-function intolerance opens new avenues for understanding neurodevelopmental disorders and refines the conceptual framework of TAU-associated disease mechanisms beyond toxic gain-of-function.

## Introduction

TAU is a major microtubule-associated protein (MAP) and regulates microtubule stability in the human brain similar to the other major MAPs MAP1A, MAP1B, and MAP2. The TAU protein is a key driver of the neurodegeneration observed in Alzheimer’s disease and related tauopathies, with deposition of intracellular TAU aggregates as the histopathological hallmark (Langerscheidt et al., 2024; Zempel, 2024), but a crucial role of TAU in human brain development has not been established. Alternative splicing of the TAU-encoding *MAPT* gene results in the expression of peripheral “big TAU” isoforms, and six human brain-specific isoforms, only one of which, the 0N3R-isoform, is expressed in the fetal brain (Hefti et al., 2018; Buchholz and Zempel, 2024).

## Search Strategy

Studies mentioned in this review were searched on PubMed using the combination of *MAPT* and gnomad or *MAPT* and “loss of function.” All years were included in the search.

## TAU Isoforms and Their Role in Disease

Apart from its microtubule binding function, non-canonical physiological and pathological functions of TAU are debated and still not fully elucidated. Besides its role in driving disease pathology in aging-associated disease, TAU is a key regulator of axonal microtubule stability and mediates axonal transport, growth, synapse formation, and other microtubule-associated processes. The six TAU isoforms expressed in the adult human brain originate from alternative splicing of exons 2, 3, and 10 of the *MAPT* gene (Goedert et al., 1989; Andreadis, 2012). The isoforms differ in the number of N-terminal inserts (0N, 1N, or 2N) and the C-terminal repeat (R) number (3R or 4R). During development, the isoform composition of the human brain switches from exclusively 0N3R to the presence of larger TAU isoforms, and results finally in about equal ratios of 3R to 4R TAU isoforms (Bullmann et al., 2009; Trabzuni et al., 2012). Notably, in the adult human brain 1N-TAU isoforms (1N3R and 1N4R) account already for 50% of the whole Tauom, while 2N-TAU isoforms are the least expressed isoforms (5%–10% of Tauom). The significance of the TAU isoform expression ratio for neuronal health is further underscored by mutations in the *MAPT* gene, which affect the splicing of TAU isoforms: e.g., reduction of 3R *versus* 4R-TAU expression is associated with frontotemporal dementia with parkinsonism-17 (Goedert et al., 1995; Buée and Delacourte, 1999). Tauopathies are classically also categorized by the isoforms present in neurofibrillary tangles composed of TAU protein: Only 4R TAU isoforms accumulate in the brains of progressive supranuclear palsy and cortical basal degeneration patients, mainly 3R TAU isoforms are present in argyrophyil grain disease and Down syndrome accumulations, but neurofibrillary tangles positive for both, 3R and 4R TAU isoforms, can be found in the brains of Alzheimer’s disease patients. In contrast, peripheral TAU (called “Big TAU” due to its size ~2-fold as big as the central nervous system [CNS]-isoforms), is not associated with disease, and not expressed in the brain. It is also not expressed in the CNS, except for neurons that project to the PNS, such as spinal motor neurons, retinal ganglion cells, and many cranial nerve neurons, all of which express Big TAU as they mature into adulthood. The region of Big TAU corresponding to exon 5 (classically 4a) has almost no homology to known proteins and almost no putative phosphorylation sites, suggesting that it arose evolutionarily from an intron of another protein. Big TAU has a lower propensity to form toxic aggregates and fibrils, in line with its lack of disease association compared to brain TAU (Fischer and Baas, 2020). While the functions of the individual isoforms are underexplored, this implies a major role of the brain-specific TAU isoforms in disease pathogenesis.

## TAU Mutation and Lessons from Transgenic Mice

Human *MAPT*-mutations associated with TAU-related disease mainly show increased aggregation propensity. A pleiotropy of mouse models expressing human mutant TAU (also and commonly in addition to endogenous mouse Tau) recapitulate human disease, in agreement with a toxic gain-of-function (i.e., increased aggregation propensity)-based disease mechanism, but with obvious limitations (for critical discussion see [Sahara and Yanai, 2023]), usually resulting in the presence of insoluble neurofibrillary tangles disrupting neuronal function.

Conversely, murine *Mapt*-knockout (KO) mice from different laboratories all resulted in viable, fertile, and principally healthy mice, with only minor neurological deficits. As *Mapt*-KO mice age, they develop mild motor deficits, such as slower performance in tasks such as the pole test and Rotarod, and reduced spontaneous movement in open field tests. *Mapt*-KO mice also tend to exhibit increased anxiety-like behaviors in standard behavioral assays. In terms of learning and memory, they show impairments in associative learning, particularly in fear conditioning tasks, which suggests dysfunction in the hippocampus and amygdala. At the synaptic level, these mice often have deficits in synaptic plasticity, including impaired long-term potentiation and reduced levels of brain-derived neurotrophic factor in the hippocampus. Most studies find, however, that spatial memory remains largely intact, and find no significant anatomical abnormalities in the brains of these mice, both of which are likely due to compensatory upregulation of other microtubule-associated proteins during development (reviewed in Ke et al., 2012).

## Metric Analysis and Loss-of-Function of *MAPT* in Humans

Thus, while gain-of-function is an established disease mechanism for *MAPT*-related disease, loss-of-function (LoF) is not. Microdeletions only encompassing the gene *MAPT* have not been described. Microdeletion at 17q21.31 (where *MAPT* is located) associated with disease usually encompasses also the gene *KANSL1*, haploinsufficiency or heterozygous pathogenic mutations of which are sufficient to cause Koolen-de-Vries Syndrome (KdVS), a rare intellectual disability syndrome with dysmorphic features (Koolen et al., 2006; Shaw-Smith et al., 2006). Notably, the severity of the phenotype of KdVS is reported to be similar in patients with a pathogenic SNV in *KANSL1* and in patients with a heterozygous deletion that includes *MAPT* (Koolen et al., 2016). As (i) *MAPT* heterozygous deletions do not add significantly to the intellectual disability of KdVS, (ii) *MAPT* heterozygous deletions are in principle compatible with life, and (iii) several murine *Mapt*-KO models are basically healthy, there was up to now no hint towards intolerance against LoF of *MAPT*.

**Figure 1:**
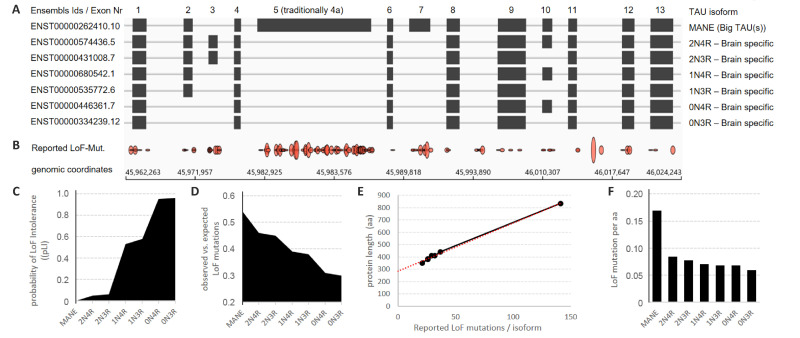
*MAPT* loss-of-function mutations (LoF-Mut) are underrepresented in the only TAU isoform present in the developing brain, but not in other brain isoforms or peripheral TAU. (A) Graphical depiction (adapted from gnomad v4.0.0, selected isoforms shown) of the MANE transcript (upper row), the two most mature isoforms (2N-TAUs, representing only ~10% of the TAUom of the brain, rows 2 and 3), and the four main brain isoforms (rows 4–7), of which the 0N3R isoform is the only one present in the developing brain. (B) LoF -Mut from ~725.000 NGS-datasets (exomes, only, unbiased selection, upper part), and the genomic coordinates (lower part) of chromosome 17. (C) Plots of the pLI score of the MANE-transcript (here representative for peripheral TAU) and the six individual brain isoforms. (D) Plots of the observed *versus* expected number of LoF -Mut in TAU. Note that the MANE transcript includes the other transcripts (see A1). (E) Reported LoF -Mut *versus* protein lengths and linear regression analysis indicate a ~300aa part of TAU where LoF -Mut may not be tolerated. (F) LoF -Mut load is ~3-fold higher in the MANE-transcript than in the 0N3R isoform, and ~5 higher in the MANE-transcript part without 0N3R-TAU exons. Note that 0N3R isoform is contained in the MANE-transcript. All data are available at https://gnomad.broadinstitute.org/.

This was also in line with early versions of gnomad, e.g., in version 2.1.1, this database comprised ~140.000 exomes and genomes and predicted the probability of intolerance to LoF (pLI) for the Matched Annotation from the NCBI and EMBL-EBI (MANE) transcript (transcript ENST00000262410.10, see [Morales et al., 2022] for a description of MANE) of *MAPT* to be 0.01 (ranging from 0, completely tolerant, to 1, completely intolerant). Also using the very recent and significantly improved release of gnomAD v4.0.0, which includes exome and genome data from **~**800,000 total individuals (Karczewski et al., 2020), again overall *MAPT* pLI is 0.00, in line with the above mentioned evidence of *MAPT* being tolerant to LoF. Other commonly used scores such as the HI- or the RVI-score are conflicting (0, meaning tolerant, and –0,76, meaning intolerant to haploinsufficiency/LoF, respectively), and are here of little help because they are not isoform-specific or rely on the whole Gene/MANE transcript. However, the MANE transcript is a theoretical transcript, which is in the case of *MAPT* for a neuronal/neurological protein biologically of little relevance: It is possibly only expressed in peripheral/adipose tissue, but not in the CNS/brain (see also above), and the closest neuronal protein correlate (Big TAU) is only expressed in the PNS and non-brain tissues (Fischer and Baas, 2020). In contrast, during human (and other mammalian species) brain development, the isoform exclusively expressed is 0N3R-TAU (transcript ENST00000334239.12). For this isoform, the pLI-score is 0.96 (hence close to 1, which would mean predicted to be intolerant to LoF). The pLI-score of the other CNS-isoforms (all of which are only expressed weeks to months after birth, depending on the species and the isoform), all of which are based on and contain completely the 0N3R-isoform (**Figure 1A**), ranges between 0.95 for 0N4R-TAU, which only contains 1 exon more than 0N3R, and 0.05 for 2N4R, which contains three more exons than 0N3R TAU (**Figure 1A** and **C** and **Table 1**). This is in line with the scarcity of LoF mutations in the regions that make up 0N3R-TAU, with an observed over-expected (o/e) of 0.3, which demonstrates a strong natural selection against LoF mutations in 0N3R-TAU. This is in gross contrast to the LoF mutational load in exons 5 (traditionally 4a) and 7, which carry an abundance of LoF mutations, but are not present (spliced out) in the human brain isoforms (**Figure 1A**, **B**, and **D**). Interestingly, even though TAU is an intrinsically disordered protein, the Missense Z-score is surprisingly high (ranging from 2.8–3.1 for the brain isoforms putting them in range also for missense intolerance *versus* 2.4 for the MANE transcript being more tolerant), while the Synonymous Z-score is unsuspicious (**Table 1**).

**Table 1:** MANE-transcript (representative of peripheral TAU isoforms) properties and brain-specific isoforms for loss of function (LoF) mutations

TAU isoform	aa	pLI	LoF o/e	LoF-mut load	LoF-mut load/aa	Z scores: Missense	Z-score: Synonymous
MANE	833	0.00	0.54 (0.41–0.72)	141	0.29	2.4	0.2
2N4R	441	0.05	0.46 (0.31–0.68)	37	0.42	3	1.18
2N3R	412	0.06	0.45 (0.31–0.68)	32	0.53	3.1	1.2
1N4R	410	0.53	0.39 (0.26–0.61)	29	0.50	2.96	1.37
1N3R	383	0.58	0.38 (0.25–0.61)	26	0.84	3.07	1.39
0N4R	381	0.95	0.31 (0.19–0.53)	26	0.90	2.82	1.31
0N3R	352	0.96	0.3 (0.18–0.53)	21	1.00	2.93	1.33

Protein length (aa), probability of LoF-Intolerance (pLI), observed over expected (o/e) LoF mutations, total LoF mutation load (LoF-mut load) over the whole isoform, LoF mutation load normalized to the protein length of the respective isoform (LoF-mut load/aa), and missense and synonymous Z-scores.

The high pLI score strongly hints towards the 0N3R-TAU isoform, as the only TAU isoform present in the developing brain, as being essential for proper brain development. Linear regression analysis of the six brain isoforms and the MANE transcript suggests mutation intolerance of ~285aa of the 353aa of the 0N3R TAU isoform (**Figure 1E** and **F**). This LoF-intolerance puts 0N3R-TAU in line with the other major brain MAPs (i.e., MAP1A, MAP1B, and MAP2), all of which are intolerant to LoF (with a pLI-score of 1). MAP1A and MAP2 are currently not associated with a human disease, possibly because of incompatibility with life. *MAP1B* is associated with i.a. periventricular nodular heterotopia type 9 (PVNH9), which includes malformation of cortical development, typical of brain microtubule impairments. Of note, even homozygous knockouts for murine *Map1a* (Liu et al., 2015), *Map1b* (Pangratz-Fuehrer et al., 2005), or *Map2* (Teng et al., 2001) are viable and show no or little cortical developmental abnormalities. It is hence not surprising that murine *Mapt*-KO mice also show only subtle neurologic defects as reported before (Gonçalves et al., 2020), yet human brain TAU protein could still be essential for proper human brain development.

## Lessons from Human *MAPT* Knockout Neurons

We have recently created human *MAPT*-KO induced pluripotent stem cells using Crispr-Cas9 and differentiated them into cortical neurons. While in mice upregulation of other MAPs in response to knockout of murine Tau was observed, no such upregulation of a single MAP was observed in human Neurons in response to KO of human TAU protein, assayed e.g., by specific immunobased assays for MAP1A/B. Also using proteomics, RNAseq, and immunostainings, we could not identify compensatory upregulation of single other MAPs. However, genes associated with the microtubule bundle show an exceptionally high fold enrichment, indicating that this cellular component is disproportionately affected by the loss of TAU protein. Other neuronal structures, such as dendritic spines, neuron spines, synapses, axons, and postsynaptic densities, also display notable enrichment, though to a much lesser extent. This pattern suggests that the absence of *MAPT* primarily disrupts microtubule organization but also has broader effects on neuronal architecture and synaptic structures. Overall, these findings highlight the central role of TAU in maintaining microtubule stability and suggest that its loss leads to widespread changes in the expression of genes crucial for neuronal structure and connectivity (Buchholz et al., 2025). In contrast to murine *Mapt*-KO primary neurons (which showed neurite outgrowth deficits only 1–2 days after plating (Dawson et al., 2001), but no later neurite defects were reported), human neurons devoid of TAU protein showed neurite outgrowth deficits after 7 days, shortening of the axon-initial-segment, and a strong trend to hyperexcitability. Reintroduction of any of the six human brain-specific TAU isoforms could rescue this deficit (Buchholz et al., 2025), indicative of the ability of all the isoforms to compensate for each other at least in development. While every cell culture system comes with limitations (e.g., biased neuronal subpopulations, lack of other brain cells, and lack of vasculature), these cells are currently one of the best proxies we have for studying the human brain. In sum, these experiments could explain that splice mutations resulting in the presence of another brain isoform may well be tolerated, but not other LoF mutations that result in the absence of any isoform during crucial developmental periods.

We reason that LoF mutations in the 0N3R isoform impact human brain development sufficiently to result in negative selection, e.g., due to intellectual disability. As *MAPT* deficiency does apparently not impact the severity of KdVS in humans, we must assume that LoF mutations in 0N3R-TAU result in mild-moderate intellectual impairments, and as it is significantly only expressed in the brain, no dysmorphic features. As mice deficient in TAU display resistance to hyperexcitability, but human neurons showed a trend towards increased excitability, in sum increased susceptibility to epilepsy should not be expected, but can also not be ruled out. We hence postulate a novel intellectual disability syndrome with mild-moderate disability without increased susceptibility to epilepsy, and no dysmorphic features, for loss of or reduced function mutations of TAU. Naturally, this assessment may vary depending on the human population, gnomad database is biased towards exomes/genomes of European descendants (77%).

## Conclusions and Open Questions

There are implications for other TAU-associated diseases. This analysis implies that depletion (as would be the case in e.g. RNAi- or antibody-based approaches for Alzheimer’s disease and related tauopathies) of 0N3R-TAU may have subtle neurological side effects, as in humans the 0N3R-isoform is continuously expressed, and may be implicated in adult neurogenesis/neurodevelopment. This should be taken into consideration for TAU-suppression-based therapies for neurodegenerative disease, i.e. therapeutic strategies should focus on specific (pathologic or pathology-driver) subspecies of TAU. In passive immunotherapy studies using the antibodies gosuranemab and tilavonemab patients with early Alzheimer’s disease or progressive supranuclear palsy showed worse cognitive outcomes in some studies, suggesting potential harm rather than benefit (Imbimbo et al., 2023), and antisense oligonucleotides were reported to result in ventricular enlargement and in elevated neurodegeneration markers in the CSF (Mummery et al., 2023). Hence, while TAU is certainly a key driver of disease, it does have physiological functions, and TAU reduction strategies should be focussed on disease-driving subspecies.

Future studies must identify suitable subspecies of TAU that drive pathology, but do not essentially contribute to the physiological functions of TAU, naturally how to target them, and possibly also in more advanced model systems such as human brain organoids or primates. Selective targeting of aggregated or oligomerized TAU, posttranslationally modified TAU, and splice isoforms are all obvious selection criteria, but this list must be expanded and possibly combined, to be able to suppress pathological TAU without affecting brain function. From a genetic disease perspective, it is currently unclear what the exact phenotype of TAU haploinsufficiency may be, as it is e.g., unresolved whether lack of TAU expression in the peripheral or enteric nervous system, where also some TAU is expressed, would result in appreciable symptoms, e.g., (enteric) neuropathy. Lastly, environmental and epigenetic modulation is insufficiently studied. In sum, while there is now solid evidence both from human model systems and databanks that TAU depletion results in notable changes in neuronal physiology and shows intolerance against loss of function mutations, future studies will have to address the actual clinical significance and the precise physiological roles of TAU.

## Data Availability

*Not applicable*.
